# Validation of the mobile verbal learning test: Illustration of its use for age and disease‐related cognitive deficits

**DOI:** 10.1002/mpr.1859

**Published:** 2020-11-07

**Authors:** Raeanne C. Moore, Emily W. Paolillo, Erin E. Sundermann, Laura M. Campbell, Jeremy Delgadillo, Anne Heaton, Joel Swendsen, Colin A. Depp

**Affiliations:** ^1^ Department of Psychiatry University of California San Diego San Diego California USA; ^2^ SDSU/UC San Diego Joint Doctoral Program in Clinical Psychology San Diego California USA; ^3^ Advancing Diversity in Aging Research (ADAR) Program San Diego State University San Diego California USA; ^4^ National Center for Scientific Research University of Bordeaux EPHE PSL Research University Bordeaux France; ^5^ VA San Diego Healthcare System San Diego California USA

**Keywords:** ambulatory assessment, ecological momentary assessment, memory, mobile health, smartphones

## Abstract

**Objective:**

We developed a mobile cognitive test of verbal learning and memory, the mobile verbal learning test (mVLT), to allow for brief, repeated and portable delivery of a 12‐item list learning test through a smartphone. This study examined the psychometric properties of the mVLT among older persons with and without human immunodeficiency virus (HIV).

**Methods:**

Sixty‐eight persons with HIV and 36 HIV‐negative individuals (aged 50–74) completed three trials of the mVLT on a smartphone once daily for 14 days. A different word list was administered each day.

**Results:**

Participants completed 80% of the 14 mVLT administrations, equating to 1166 valid and complete mVLTs. Neither adherence nor mean mVLT total score (number correct in 3 recall trials) differed by HIV status. No practice effects from repeated mVLT administration were observed, and there were moderately strong correlations of mVLT performance with performance on the in‐lab version of the task and with traditional cognitive assessments of cognitive processes contributing to memory. We found evidence of within‐person learning across mVLT trials, with persons with HIV demonstrating less learning from trials 1 to 3 compared to HIV‐negative participants.

**Conclusions:**

The mVLT is a valid method to assess learning in the real world in older adults with and without HIV.

## 1 INTRODUCTION

A host of psychiatric and medical conditions are characterized by cognitive deficits that have important clinical consequences for functioning, medication adherence and quality of life. In this study, we focus on cognition in the context of human immunodeficiency virus (HIV) infection. HIV targets the central nervous system within days after infection, which can lead to neurological, behavioural and cognitive complications (Brew, Sidtis, Petito, & Price, 1988; Grant et al., 1989; McArthur, 1994). The advent of combined antiretroviral therapy (cART) led to a decrease in the rates of HIV‐associated dementia; however, mild neurocognitive deficits persist in approximately 45% of individuals with HIV/acquired immunodeficiency syndrome (AIDS). Older persons with HIV (PWH), in particular, are at increased risk for HIV‐Associated Neurocognitive Disorders (HAND), as well as increased risk for Alzheimer's disease (AD) and its precursor, mild cognitive impairment. Although these HIV‐associated neurocognitive deficits are observed in multiple cognitive domains, deficits are most common in the domains of episodic learning and memory and executive functions in the post cART era (Heaton et al., 2011). Detecting these deficits are challenging given that conventional neuropsychological assessments are costly, time‐intensive and impacted by stressors related to being in a clinical care environment. Furthermore, cognitive performance at any one time point can vary according to daily rhythms, environmental stressors, fatigue, emotion and other state‐dependent factors (Hess, Popham, Emery, & Elliott, 2012; Metternich, Schmidtke, & Hull, 2009; Schmidt, Collette, Cajochen, & Peigneux, 2007; Tollenaar, Elzinga, Spinhoven, & Everaerd, 2009).

Self‐administered, repeatable smartphone‐based mobile cognitive tests administered within an Ecological Momentary Assessment (EMA) paradigm (which we are referring to, collectively, as Ecological Momentary Cognitive Tests [EMCTs]) can overcome many of these challenges (Moore, Swendsen, & Depp, 2017). Still an emerging area of research, there is dearth of literature on the psychometric properties of mobile cognitive tests, although see (Moore et al., 2017, 2020; Sliwinski et al., 2016; Weizenbaum, Torous, & Fulford, 2020). While they are not a replacement for gold‐standard neuropsychological testing, mobile cognitive tests have the capability of measuring cognitive performance in one's natural environment and can be used to monitor cognitive health over time. Additionally, mobile cognitive tests allow for examination of time‐varying correlates of neurocognitive performance, such as mood variability and everyday functioning behaviours, as well as intra‐individual cognitive variability (IIV), a topic gaining more attention in the cognitive aging (e.g., Rutter, Vahia, Passell, Forester, & Germine, 2020) and dementia literature. For example, studies have shown that IIV may be indictive of early neurodegenerative processes of AD and predictive of incident mild cognitive impairment and AD (Christ, Combrinck, & Thomas, 2018; Gleason et al., 2018; Kay et al., 2017). As an example of how mobile cognitive tests can be used in clinical research, Allard et al. (2014) found that neuroimaging markers were related to mobile cognitive tests of memory performance but not to conventional laboratory‐based memory test scores. These findings may be attributable to a reduced margin of error associated with mobile cognitive tests, in that repeated testing may produce more reliable scores than one‐time administrations of traditional neuropsychological assessments. Moreover, information about behaviours, symptoms and contexts simultaneously acquired through EMA offers powerful opportunities to identify the daily life predictors (e.g., affect, physical activity and socialization) and consequences of changes in cognitive performance (Allard et al., 2014; Campbell et al., 2020; Swendsen, Schweitzer, & Moore, 2017; Weizenbaum et al., 2020). For example, among older PWH, cognitive impairment is an established precursor to daily functional impairments (Thames et al., 2011; Vance, Fazeli, & Gakumo, 2013; Vance, Wadley, Crowe, Raper, & Ball, 2011), yet functional impairments are often observed in persons with normal cognition, and vice versa (Blackstone, Moore, Heaton et al, 2012; Heaton et al., 2004); EMCTs can help tease apart this complex relationship. Overall, among those at‐risk for cognitive impairment, including older PWH, EMCTs may, therefore, improve our ability to identify cognitive change in the early stages of decline when treatments and interventions are most effective and can be best implemented.

We developed a mobile cognitive test of verbal learning and memory, called the mobile verbal learning test (mVLT), to allow for brief, repeated and portable delivery of a 12‐item list learning test through a smartphone. Herein, we provide data on the validity of the mVLT among a sample of both HIV‐infected and uninfected participants. Participants were administered three trials of the mVLT once a day for 14 days. A different list was administered each day. We examined practice, fatigue and learning effects and assessed potential interactions with HIV status. Second, we compared aggregate mean scores on the mVLT to scores with a laboratory‐based, paper‐and‐pencil version of the mVLT and with previously validated, clinical neuropsychological tests (including memory) as well as to socio‐demographic factors related to cognitive performance (age, sex, race/ethnicity and education). Impairments in learning are a defining feature of HAND in the cART era (Heaton et al., 2010), therefore we hypothesize that HIV‐uninfected participants would show greater learning across the three‐daily trials, and across study day, on the mVLT relative to HIV‐infected participants. Furthermore, we hypothesized that mVLT scores would be moderately associated with scores on an adapted laboratory version of the mVLT and standardized laboratory‐based memory tests, as well as with scores on clinical tests that assess cognitive processes contributing to memory (i.e., processing speed, attention and executive function), although the strongest association would be with memory tests. Lastly, we hypothesize that poorer performance on the mVLT would be associated with older age, male sex, less years of education, less cognitive reserve (scores on the Wide Range Achievement Test‐4 [WRAT‐4]) and greater depressive symptoms, regardless of HIV status. Evidence has demonstrated depression is an early sign of a neurodegenerative process, as well as a risk factor for conversion from mild cognitive impairment to AD (Diniz, Butters, Albert, Dew, & Reynolds, 2013; Lauriola et al., 2018; Xu et al., 2018), hence our hypothesis that greater depressive symptomatology would be associated with poorer mVLT performance, regardless of HIV status.

## 2 METHODS

### 2.1 Participants

Participants (68 persons with HIV and 36 HIV‐negative individuals) were enrolled for this study through the HIV Neurobehavioral Research Program (HNRP) at the University of California, San Diego (UCSD) between 2016 and 2019. Recruitment was conducted in one of two ways: from the participant pool at the HNRP or through community‐based recruitment (e.g., HIV clinics, flyers). If a participant had completed a neuropsychological evaluation at the HNRP within the past 6 months, in‐person neuropsychological testing was not repeated and the data were used for this study visit. The 6‐month window for recent neuropsychological testing was selected to minimize participant burden, as changes in cognition occur slowly over time and are unlikely to change in 6 months. Inclusion criteria encompassed HIV‐infected and uninfected individuals aged 50 years or older, with ability to provide written informed consent and English fluency. Exclusion criteria were: diagnosis of a non‐HIV neurological disorder, serious mental illness, head injury with loss of consciousness >30 min or indication of a severe learning disability (standard score of <70 on The WRAT‐4 Edition Reading (Wilkinson & Robertson, 2006). Participants with a positive alcohol breathalyzer or urine toxicology for illicit substances on the day of in‐person testing (with the exception of cannabis products) were rescheduled. The UCSD Institutional Review Board approved all study procedures prior to protocol implementation. All participants demonstrated decisional capacity to provide written, informed consent (Jeste et al., 2007).

### 2.2 Measures and procedure

Participants completed a comprehensive neuromedical and neurobehavioral baseline visit, followed by a 14‐day period of EMCT. After the at‐home assessment, participants returned to the HNRP for a follow‐up visit. Participants were not co‐enrolled in intervention studies during the study period. Participants were compensated for in‐person assessments ($15/hour, which is the standard pay rate for research participation at the HNRP) as well as for each mVLT test they completed ($1/test).

### 2.3 Baseline visit

Neuromedical and neurobehavioral assessments were administered to participants at the baseline visit. An HIV/HCV antibody point‐of‐care rapid test (Miriad‐MedMira™) was administered to all participants to assess HIV serostatus and confirmed by the Western Blot Test. Demographics, employment status and other indicators of socioeconomic status were collected via self‐report surveys. Among PWH, self‐report data were obtained regarding HIV characteristics, such as estimated duration of infection, nadir CD4 and antiretroviral medications, although use of antiretroviral medications was not required for participation. Viral load detectability (<50 copies/ml) and current CD4 count was measured in blood plasma. Psychiatric and substance use disorders were determined via the Composite International Diagnostic Interview (CIDI, version 2.1 “Composite International Diagnostic Interview (CIDI, version 2.1) [computer program],” 1997), a computer‐assisted structured interview. Current depression was assessed with the Beck Depression Inventory‐II (BDI‐II; Beck, Steer, & Brown, 1996), in which higher scores indicate greater depressive symptomatology.

Participants completed the standard HNRP comprehensive neuropsychological test battery (see Table 1), which includes seven domains known to be affected in PWH (verbal fluency; executive function; processing speed; learning; delayed recall; working memory and complex motor skills) and has been previously described (Heaton et al., 2011). Raw scores from the neuropsychological tests are converted into practice‐effect corrected, normalized scaled scores and averaged per domain to obtain domain scaled scores (SS, Mean = 10, SD = 3; Cysique et al., 2011). We also converted raw scores to demographically adjusted T‐scores, which are then converted into continuously distributed deficits scores ranging from 0 (corresponding to a T‐score >39; no impairment) to 5 (T‐score <20, severe impairment). These scores are averaged to derive a global deficit scores (GDS) to determine cognitive impairment (impairment GDS≥0.5; Blackstone, et al. 2012; Carey et al., 2004). Estimated cognitive reserve was measured using the WRAT‐4 Reading subtest that was administered at the screening visit. Of the individual tests administered, performance on the 12‐item Hopkins Verbal Learning Test‐Revised (HVLT‐R; Benedict, Schretlen, Groninger, & Brandt, 1998) was of particular interest in this study to evaluate convergent validity. Additionally, we created a lab‐based version of our mVLT, which was administered at the baseline visit. Participants were provided a sheet of paper with a list of 12 words and were instructed to review the words for 30 s. At completion of the time limit, the examiner removed the list of words and participants were asked to recite as many words as they could remember. Participants completed three trials of the lab‐based mVLT. It should be noted that we did not implement this lab‐based version until the study was underway, so 21 participants did not receive this task.

**TABLE 1:** HIV neurobehavioral research program neuropsychology battery

Verbal Fluency	Learning
Controlled oral word association test (FAS)	Hopkins verbal learning test‐revised (total learning)
Category fluency test (“animals” and “actions”)	Brief visuospatial memory test‐revised (total learning)
**Executive function**	**Recall**
Wisconsin card sorting test (computerized 64‐cards)	Hopkins verbal learning test‐revised (delayed recall)
Trail making test part B	Brief visuospatial memory test‐revised (delayed recall)
Stroop color and word test (interference score)	**Working memory**
**Speed of information processing**	WAIS‐III letter‐number sequencing
WAIS‐III digit symbol	Paced auditory serial addition task
WAIS‐III symbol search	**Complex motor skills**
Trail making test part A	Grooved Pegboard test (dominant and non‐dominant)
Stroop color and word test (color trial)	

Abbreviation: HIV, human immunodeficiency virus.

Following the lab‐based assessments, participants were provided with a password protected Samsung Galaxy S 4.2 YP‐GI1 8GB smartphone with 4G Android Operating system for the duration of the study. The Galaxy Player 4.2 has a 4.2″ IPS display at 800 x 480, 1 GHz processor, using Android 2.3.6 Gingerbread OS. Our software application operated without an active wifi connection, and data were stored locally on the device. Participants were provided with a 20–30 min training session on using the study smartphone and responding to EMCT prompts and given a smartphone operating manual to take home. They were asked to keep the study phone with them at all times, in addition to their personal smartphone (if applicable). The non‐study related functions of the phone were locked; thus, it was usable for the purposes of the study.

### 2.4 Smartphone tracking: 14‐day EMCT

**FIGURE 1:**
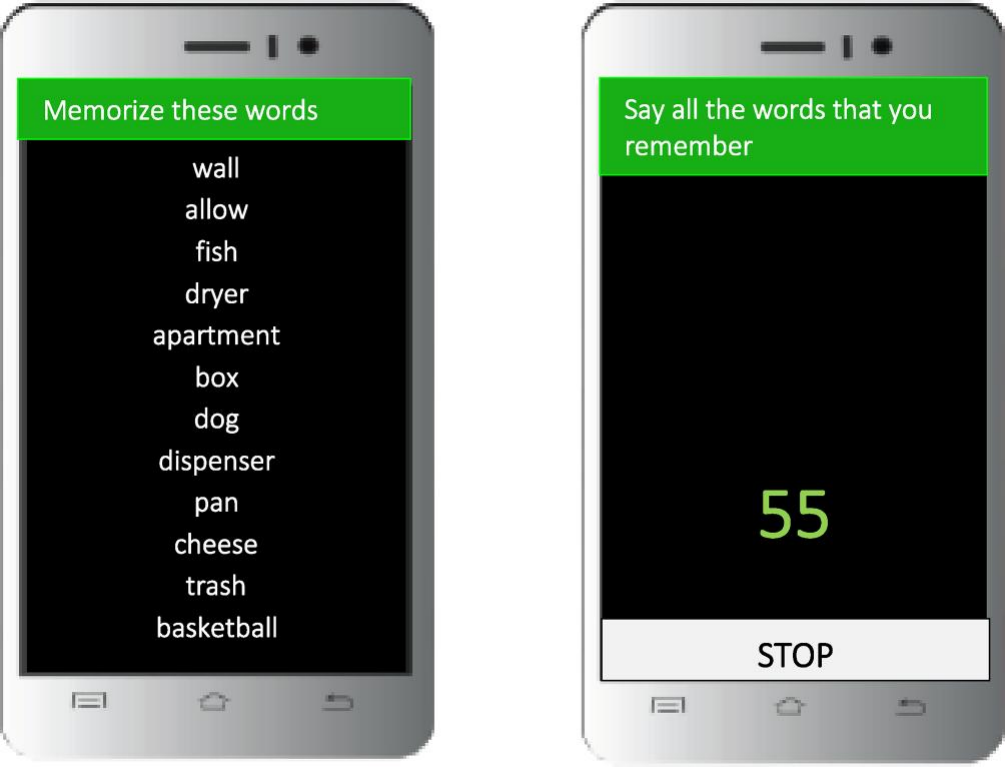
Sample screenshot of the mVLT. Please note that, for test security, the figure includes sample words and does not include actual words from the mVLT. mVLT, Mobile Verbal Learning Test

For the 2‐week study period, participants were administered four surveys per day, approximately 3 hours apart, on a schedule customized to their preferred sleep/wake schedule. The mVLT was administered at the end of one of the surveys each day (sample screenshot of the mVLT, Figure 1). The timing of the administration was counter‐balanced so the mVLT was administered at different times of the day. The same version was administered on the same day to all participants (i.e., list 1 was administered on day 1, list 2 was administered on day 2, etc.). Participants also completed a test of executive function, the mobile color‐word interference test (mCWIT; Moore et al., 2020), once daily. The two mobile cognitive tests were never administered at the same time to reduce participant burden. Modelled after traditional neuropsychological list learning tasks, the mVLT consists of 12 semantically unrelated words. We created 14 different word lists, one for each day, using the SUBTLEX(US) database (http://www.lexique.org/?page_id=241). This database contains word frequencies for 50 million words. The words are based on English‐US movies and TV series subtitles, which have been found to be a better source of word frequencies than written text (Brysbaert & New, 2009). First, we eliminated proper nouns and curse words from the database, then we wrote a script to select a random subset of 12 words from this database based on a set of user‐defined parameters, including: word length min/max, part of speech, minimum threshold for word frequency and excluding plural forms (i.e., nouns ending in ‘s’ where it is not preceded by ‘I’ or ‘u’, or nouns ending in ‘ae’) and tense exclusions (i.e., any verbs ending in ‘ed’ or ‘ing’). The selection criteria were limited such that each list was matched for word frequency. We also created a fifteenth list for the lab‐based VLT.

Once prompted to start the mVLT, participants were presented with a list of words for 30 s. Then a screen with instructions appeared on the smartphone and participants were asked to say aloud how many words they recalled, in any order. Participants were given up to 1 min to recall words for each trial but could choose to select ‘Done’ on the screen if they completed the task in less than one minute or did not want to wait until the time ran out. Once the first trial was completed, a second trial of the same list was performed, followed by a third trial. Responses were audio recorded on the smartphone. Each audio file was listened to and scored independently by two trained raters. All words that were said by participants were transcribed, and raters documented the number of correct responses, number of intrusions, number of repetitions and potential cheating. Potential cheating was determined if a voice other than the participants was making the responses or heard providing help with the test content, if it was highly suspected the participant wrote down the words (for instance, when a participant said all the 12 words quickly and in order and this represented a deviation from their normal performance), or other issues that may call into question the validity of the test data. If there were discrepancies in ratings between the two raters, a third independent rater would listen to and score the audio file.

### 2.5 Follow‐up visit

After the 2‐week EMCT period was concluded, participants came back to the HNRP to return the smartphone and complete a feedback questionnaire.

### 2.6 Statistical analyses

HIV group differences in demographics and clinical factors were examined using independent *t*‐tests (or non‐parametric Wilcoxon tests) for continuous variables or chi‐squared analyses (or non‐parametric Fisher's exact tests) for categorical variables. Practice effects (i.e., improvements in mVLT total score over the 14‐day study period) and fatigue effects (i.e., likelihood of missing mVLT over the 14‐day study period) on the mVLT were examined using linear and logistic‐mixed effects models, respectively, to determine the within‐person effect of study day on each of those two outcomes. Additional mixed effects models examined whether practice and fatigue effects differed by HIV status by including an interaction between HIV status and study day. Convergent validity was examined in the following ways. First, Pearson correlations were used to determine strength of the relationships between average mVLT performance and laboratory‐based cognitive performance. Next, we examined associations between demographic and HIV disease‐related factors with mVLT performance and using independent *t*‐tests or Pearson correlations as appropriate. Last, we examined within‐person learning across the three mVLT trials using linear mixed effects models. In order to understand whether significant improvements in performance were occurring from both trial 1 to trial 2 and trial 2 to trial 3, trial number was set as a factor variable with trial 2 as the reference. Two additional linear‐mixed effects models examined whether within‐person learning differed by: (1) HIV status (by including an interaction between HIV status and trial) and (2) study day (by including an interaction between study day and trial). For these additional linear‐mixed effects models, trial 1 was set as the reference. All analyses were performed using R, version 3.5.0.

## 3 RESULTS

### 3.1 Sample characteristics

Demographic and clinical characteristics by HIV status are displayed in Table 2. There was a higher proportion of male participants among PWH compared to that of HIV‐participants (*p* < 0.01); however, all other demographic factors were comparable (*p*s > 0.05) between groups. Rates of smartphone ownership were high (>80%) and similar across groups (*p* > 0.05). Regarding HIV disease characteristics, our sample of PWH had well controlled viral loads, with 94% on ART and 97% with undetectable HIV plasma viral loads. PWH had higher average depressive symptom severity on the BDI‐II at their baseline visit compared to HIV‐ participants (*p* < 0.01); however, all other clinical factors (e.g., cognitive performance) were similar between groups (*p*s > 0.05). Over one‐third of the participants (36% of PWH; 37% of HIV‐ participants) had GDS scores in the impaired range.

**TABLE 2:** Demographics and clinical characteristics by HIV status

	HIV+ (*n* = 68)	HIV− (*n* = 36)	Cohen's d	Test‐statistic*	*p*‐value
Age (years)	59 (6.2)	59 (6.7)	0.01	0.04	0.96
Sex (male)	55 (81%)	20 (56%)	1.48	7.51	**<0.01**
Race/Ethnicity (non‐Hispanic White)	43 (63%)	23 (64%)	0.00	0.004	0.95
Education (years)	14 (2.5)	15 (2.5)	0.37	1.87	0.07
Employment status (employed)^a^	20 (30%)	14 (40%)	0.19	0.96	0.33
WRAT‐4 reading	102.0 (14.5)	105.6 (16.4)	0.23	1.16	0.25
Smartphone ownership (iPhone or android vs. no phone or other)	60 (88%)	30 (83%)	0.10	0.49	0.49
History of AIDS	46 (68%)	‐	‐	‐	‐
Current CD4 count	706.5 [556.3, 893.8]	‐	‐	‐	‐
Nadir CD4 count	158 [35.3, 300]	‐	‐	‐	‐
Estimated duration of infection (years)	22.5 (7.9)	‐	‐	‐	‐
On antiretroviral therapy	64 (94%)	‐	‐	‐	‐
% Detectable plasma viral load	2 (3.3%)	‐	‐	‐	‐
BDI‐II	9.8 (9.7)	3.1 (3.8)	0.99	5.04	**<0.01**
GDS‐impaired^b^	25 (36%)	13 (37%)	0.00	0.001	>0.99
Global cognition scaled score (SS)^b^	8.8 (2.0)	9.4 (1.9)	0.31	1.58	0.12
Verbal fluency SS^a^	10.3 (2.6)	11.4 (2.8)	0.38	1.93	0.06
Executive functioning SS^c^	8.3 (2.5)	9.1 (2.2)	0.30	1.54	0.13
Processing speed SS^a^	9.5 (2.3)	10.2 (2.5)	0.25	1.26	0.21
Learning SS^c^	6.7 (2.6)	7.7 (2.6)	0.34	1.75	0.08
Delayed recall SS^c^	7.0 (2.5)	7.7 (2.8)	0.27	1.38	0.17
Working memory SS^c^	9.9 (2.8)	10.3 (2.8)	0.15	0.74	0.46
Complex motor skills SS^d^	7.7 (2.7)	8.0 (2.4)	0.08	0.43	0.67
HVLT‐R total (number correct in 3 recall trials)^c^	24.6 (5.2)	25.8 (5.1)	0.22	1.13	0.26
In‐person 30‐s mVLT total score (number correct in 3 recall trials)^e^	21.0 (5.2)	24.0 (5.3)	0.44	2.18	**0.03**
Mean mVLT total score (number correct in 3 recall trials)	20.0 (4.9) [8.9–31.3]	21.1 (3.8) [14.9–28.9]	0.24	1.20	0.23

*Note:* Values are presented as mean (SD), median [IQR], or *N* (%); Bolded *p*‐values are significant at *p* < 0.05.

Abbreviations: AIDS, acquired immunodeficiency syndrome; BDI‐II, Beck Depression Inventory‐II; GDS, Global Deficit Score; HIV, human immunodeficiency virus; HVLT‐R, Hopkin's Verbal Learning Test‐Revised; SS, scaled score; WRAT‐4, Wide Range Achievement Test‐Revised.

* *T*‐tests for continuous variables; Chi^2^ for dichotomous variables.

^a^ *n* = 101.

^b^ *n* = 103.

^c^ *n* = 102.

^d^ *n* = 99.

^e^ *n* = 67.

### 3.2 mVLT adherence

One hundred and twenty trials (9%) were invalid (e.g., cheating and interruptions) and were not included in analyses. Participants had an average of 80.1% adherence to the mVLT protocol (SD = 17.2%; range = 28.6%–100%), resulting in 1166 valid and complete mVLTs among all 104 participants. mVLT adherence did not differ by HIV status (HIV+: *M* = 79.1%; HIV−: *M* = 81.9%; *t =* 0.88; *p* = 0.38) or by employment status (Employed: *M* = 81.3%; Unemployed: *M* = 79.2%; *t* = 0.59; *p* = 0.56). Better mVLT adherence was not significantly related to older age (*r* = 0.18, *p* = 0.07) or being cognitively normal (vs. cognitively impaired; *t* = −1.98, *p* = 0.05).

### 3.3 Practice and fatigue effects

**FIGURE 2:**
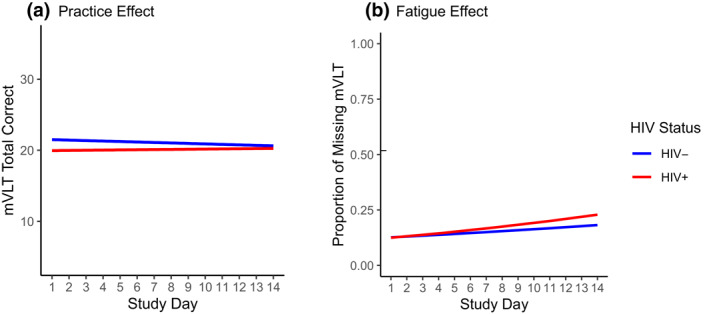
Mobile Verbal Learning Test practice and fatigue effects (Panels A and B, respectively) by HIV status. HIV, human immunodeficiency virus

In the overall sample, there was no practice effect *in total score* from repeated mVLT administration across the study period (coefficient = –0.001, *SD* = 0.023, *t* = −0.025, *df* = 1061, *p* = 0.980). There was also no difference in practice effect by HIV status (coefficient = 0.073, *SD* = 0.058, *t* = 1.257, *df* = 1064, *p* = 0.209; Figure 2 Panel A). A significant fatigue effect was observed such that there was a small increase in the likelihood of a missed mVLT over the course of the study period within persons (OR = 1.050 [per 1‐day increase], 95% CI = 1.015–1.086, *p* = 0.005). This fatigue effect did not differ by HIV status (OR = 1.023, 95% CI = 0.953–1.100, *p* = 0.524; Figure 2 Panel B).

### 3.4 Convergent validity

Correlations between average mVLT scores and laboratory‐based cognitive performance are presented in Table 3. Average mVLT performance was moderate‐to‐strongly correlated with the in‐laboratory administered mVLT and the well‐validated and commonly used HVLT‐R (*p*s ≤ 0.001). Performance on the in‐lab HVLT‐R was also significantly correlated with the in‐lab mVLT (*r* = 0.43, *p* < 0.001). In terms of composite global and domain scores from the comprehensive neuropsychological battery, average mVLT performance was most strongly related to verbal fluency, followed by global cognitive function, learning, executive functioning, delayed recall, working memory and processing speed. mVLT performance was not related to the cognitive domain of complex motor skills (*p* > 0.05).

**TABLE 3:** Correlations between the mVLT and laboratory‐based cognitive performance

Laboratory‐based tests and cognitive domains	Cohen's d	Pearson *r*	*p*‐value
*Individual Tests*			
In‐lab 30‐s mVLT^a^	1.71	0.65	**<0.001**
In‐lab HVLT‐R	1.01	0.45	**<0.001**
*Composite cognitive SS*			
Global SS	1.22	0.52	**<0.001**
Verbal fluency SS	1.28	0.54	**<0.001**
Executive function SS	1.04	0.46	**<0.001**
Processing speed SS	0.65	0.31	**0.002**
Learning SS	1.09	0.48	**<0.001**
Delayed recall SS	0.87	0.40	**<0.001**
Working memory SS	0.68	0.32	**0.001**
Complex motor SS	0.16	0.08	0.414

*Note*: Bold values indicate statistical significance.

Abbreviations: HVLT, Hopkins Verbal Learning Test; mVLT, Mobile Verbal Learning Test; SS, scaled scores; VLT, Verbal Learning Test.

^a^ *n* = 67; not all participants were given the in‐lab mVLT.

In terms of demographics, better average mVLT performance was significantly related to more years of education (*r* = 0.34, *p* = 0.001); however, mVLT was not related to age (*r* = 0.01, *p* = 0.942), sex (Men: *M* = 20.3, *SD* = 4.7; Women: *M* = 20.6, *SD* = 4.3; *p* = 0.74) or race/ethnicity (non‐Hispanic White: *M* = 20.8, *SD* = 4.2; Other: *M* = 19.7, *SD* = 5.1; *p* = 0.26). Average mVLT performance significantly related to cognitive reserve (i.e., WRAT‐4 Reading Score; *r* = 0.35, *p* < 0.001), but was not related to depressive symptoms on the BDI‐II (*r* = −0.15, *p* = 0.13). Average mVLT performance also did not differ by HIV status (HIV+: *M* = 20.0, *SD* = 4.9; HIV−: *M* = 21.1, *SD* = 3.8; *p* = 0.23). Among PWH only (*n* = 68), there were no relationships between mVLT performance and disease characteristics, including AIDS status (AIDS: *M* = 19.9, *SD* = 4.9; Non‐AIDS: *M* = 20.3, *SD* = 5.1; *p* = 0.76), duration living with HIV (*r* = −0.07, *p* = 0.57), nadir CD4 count (*r* = 0.05, *p* = 0.70), current CD4 count (*r* = −0.02, *p* = 0.89) and log_10_ plasma HIV RNA (*r* = 0.07, *p* = 0.56).

### 3.5 Learning

**FIGURE 3:**
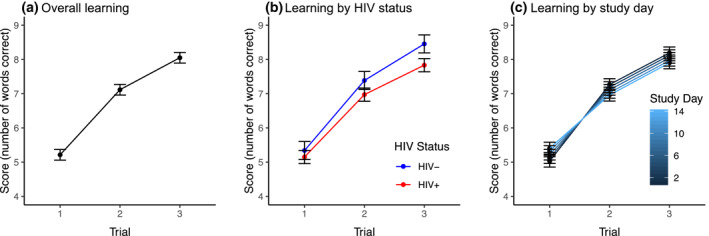
Within‐person learning across trials on the Mobile Verbal Learning Test in the overall sample (Panel A), by HIV status (Panel B), and by study day (Panel C). HIV, human immunodeficiency virus

Regarding within‐person learning on the mVLT, there was a stairstep effect of trial number, such that scores from mVLT trial 1 were significantly lower than that of trial 2 (coefficient = −1.901, *SE* = 0.066, *df* = 3392, *p* < 0.001), and scores from mVLT trial 3 were significantly higher than that of trial 2 (coefficient = 0.934, *SE* = 0.066, *df* = 3392, *p* < 0.001; Figure 3 Panel A). This within‐person learning across mVLT trials appeared to differ significantly by HIV status, such that PWH had less learning from trial 1 to trial 3 compared to that of HIV‐ participants (*p* = 0.002; Table 4; Figure 3 Panel B). Within‐person learning also appeared to differ significantly across study days, such that learning slopes (both from trial 1 to trial 2 [*p* = 0.001] and trial 1 to trial 3 [*p* = 0.002]) were steeper at the earlier study days compared to the later study days (Table 4; Figure 3 Panel C). Notably, this model revealed a conditional main effect of study day (coefficient = 0.029, *p* = 0.013) indicating that trial 1 performance improved over the course of the study.

**TABLE 4:** Results of two linear mixed‐effects models examining the moderating effects of HIV status and study day on within‐person learning across trials on the mVLT

Model examining effect of HIV Status	Coefficient	SE	*p*‐value
Intercept	5.339	0.264	**<0.001**
Trial 2 (vs. trial 1)	2.046	0.110	**<0.001**
Trial 3 (vs. trial 1)	3.114	0.110	**<0.001**
HIV status (+vs. −)	−0.190	0.326	0.561
Trial 2 (vs. trial 1) × HIV status (+vs. −)	−0.224	0.137	0.103
Trial 3 (vs. trial 1) × HIV status (+vs. −)	−0.431	0.137	**0.002**

Model examining effect of study day	Coefficient	SE	*p*‐value
Intercept	4.999	0.178	**<0.001**
Trial 2 (vs. trial 1)	2.289	0.138	**<0.001**
Trial 3 (vs. trial 1)	3.215	0.138	**<0.001**
Study day	0.029	0.012	**0.013**
Trial 2 (vs. trial 1) × study day	−0.053	0.017	**0.001**
Trial 3 (vs. trial 1) × study day	−0.052	0.017	**0.002**

*Note:* Bolded *p*‐values are significant at *p* < 0.05.

Abbreviations: HIV, human immunodeficiency virus; mVLT, Mobile Verbal Learning Test.

## 4 CONCLUSIONS

Findings from this study contribute to the literature by demonstrating acceptability and construct validity of our newly developed ambulatory learning and memory test, the mVLT, among persons with and without HIV. Main findings include: (1) excellent adherence to the study protocol, with participants completing 80% of the 14 mVLT administrations; (2) no practice effects from repeated mVLT administration across the 14‐day study period; (3) a small fatigue effect, such that adherence to the protocol diminished somewhat across the 14 testing sessions; (4) large effect sizes between mVLT performance with performance on the in‐lab version of the task as well as with traditional cognitive assessments of cognitive processes contributing to memory and (5) evidence of within‐person learning across mVLT trials, with PWH demonstrating less learning from trials 1 to 3 compared to HIV‐ participants.

Statistically significant psychometric test property differences by HIV status were not found for adherence, practice and fatigue effects, or convergent validity. The lack of group differences in aggregate mVLT performance is in‐line with the lack of group differences in lab‐based cognitive abilities, and speaks to the validity of aggregate mean scores of this ambulatory cognitive test. Interestingly, however, we did see group differences on in‐person mVLT total score (number correct in 3 recall trials) performance, such that the HIV‐ participants recalled more words than PWH. Furthermore, PWH showed less learning from trials 1 to 3 across the 14 days of ambulatory assessment. These findings suggest that the mVLT is potentially capturing subtle group differences in learning that are not observed at an aggregate mean level. Regarding the lack of practice effects, the words on the mVLT lists were not semantically related so traditional cognitive strategies (e.g., clustering) people employ for improving performance on many traditional neuropsychological list learning tests (such as the HVLT), could not be utilized. Another possible explanation for the lack of practice effects could be proactive interference from the prior administration of the mVLT, given the test‐retest interval was within 24 h.

### 4.1 Limitations and considerations for future research

This study is not without limitations that should be considered when interpreting the findings. The sample size was relatively small and the participants were a specific population; further work is needed to continue the validation of the mVLT in various neuropsychiatric populations. We did not observe relationships between the mVLT and sex among the whole sample, so we chose not to covary for sex in our models. Given there were significantly more females in the HIV‐group, and females generally do better on verbal list learning tests than males, future studies are needed with larger samples of demographically matched control participants to replicate these findings. Scalability of the mVLT is also a concern, as the audio files had to be listened to by trained raters and manually scored. At this time, to our knowledge, the quality of voice recognition software is not adequate to detect accents, various dialects, differentiate between voices and background noise or identify variations in speech patterns. Voice recognition software is improving, and, in the near future, it is likely that it will be of sufficient quality to conduct automatic scoring. We have also developed a newer touch‐response version of the mVLT, which reduces administrative burden. However, it is yet to be determined whether having participants provide verbal (vs. forced‐choice touch) responses yields comparable results, and the latter is largely a task of recognition memory. Another concern is the difficulty in identifying suspected cheating or if a participant was obtaining help from others. However, only 9% of the trials were flagged as invalid (e.g., cheating, interruptions heard on the recording) by the raters. Lastly, issues around privacy and standards around best practices for capturing mobile cognitive testing data are still being established. Before EMCT can be deployed at‐scale for research or clinical use, ethical standards and privacy policies need to be in‐place. As evidence of the need for best practices, a recent systematic review found the majority of currently available commercial‐grade app‐based cognitive assessment tools lack any form of validity data (Charalambous et al., 2020).

In sum, the capability to repeatedly administer memory tasks (and other cognitive tasks) in a person's natural environment, without concerns for practice effects, offers several advantages, such as the ability to understand the contexts in which cognition vary in everyday life. Furthermore, we found convergent validity of the mVLT with lab‐based measures, as well as evidence of learning across trials. Given that the mVLT is associated with a gold standard in‐lab memory task, there several potential applications for the mVLT and other intensively repeated mobile cognitive tasks. For one, instability in memory within individuals can be evaluated as another indicator that may herald subtle decline. Second, combination with EMA allows for more precise evaluation of day‐to‐day contextual or behavioural influences (e.g., cognitive or physical activities) on memory performance, paving the way for mechanistic research on novel risk factors or intervention targets. Third, given the host of passive digital biomarkers of cognition or risk factor that are now possible (e.g., GPS), repeated mobile cognitive testing could provide a robust platform with which to validate such biomarkers as they track level and intra‐individual variation in cognitive performance over time. Finally, while this investigation demonstrates the validity of the mVLT in older individuals with or without HIV, its pertinence to psychiatric disorders is also evident given the existence of mild to severe cognitive deficits frequently associated with schizophrenia, mood disorders, substance use disorders and a range of other conditions. The use of such tools in psychiatric samples should permit the detection of subtle but clinically relevant cognitive difficulties that may have direct implications for daily life functioning and symptom expression.

## AUTHOR CONTRIBUTIONS

Raeanne C. Moore: Designed the study, created the mVLT word lists, supervised data collection and analyses, interpreted the data, and wrote the manuscript; Emily W. Paolillo: Performed data analyses, created the tables and figures, and helped write and edit the manuscript; Erin E. Sundermann and Jeremy Delgadillo: helped write the manuscript; Laura M. Campbell: supervised data scoring and substantively edited the manuscript; Anne Heaton: data acquisition, helped create tables, and substantively edited the manuscript; Joel Swendsen: programming on mobile tasks and substantively edited the manuscript; Colin A. Depp: helped with study design and substantively edited the manuscript. All authors approved the submitted version.

## CONFLICTS OF INTEREST

The authors have declared that they have no conflicts of interest.
